# Tachycardia during Treatment with Risperidone and Paliperidone Palmitate in a Patient without Previous Cardiovascular Disease

**DOI:** 10.1155/2021/9954991

**Published:** 2021-07-16

**Authors:** Zachary Orlins, Brian Barnett

**Affiliations:** Department of Psychiatry and Psychology, Center for Behavioral Health, Neurological Institute, Cleveland Clinic, Cleveland, OH, USA

## Abstract

Here, we present the case of a patient who initiated risperidone and developed persistent tachycardia, which was exacerbated by subsequent administration of paliperidone palmitate despite treatment with propranolol. This patient's experience emphasizes the need for psychiatrists to regularly monitor vital signs after risperidone/paliperidone initiation, dosage increase, or overdose to identify and appropriately manage this potentially harmful side effect. Increasing clinician awareness of this rare side effect will help protect patients with serious mental illness who regularly rely upon these medications, particularly in their long-acting injectable formulations.

## 1. Introduction

Patients with serious mental illness often have risk factors for adverse cardiovascular outcomes, such as nicotine dependence, general neglect of health, poor diet, and limited access to health care services. Additionally, treatment with antipsychotic medications can cause metabolic syndrome [1] and QTc interval prolongation, a risk factor for the polymorphic ventricular tachycardia Torsades de Pointes (TdP) [2]. The antipsychotic risperidone and its active metabolite, paliperidone, are associated with QTc prolongation and, more rarely, TdP, as well as other forms of tachycardia such as multifocal atrial tachycardia [3] and sinus tachycardia [4, 5]. Risperidone's potential to induce tachycardia is especially prominent in cases of toxicity, with one study finding that 58% of patients who overdosed on it experienced tachycardia without dysrhythmia [2]. Sinus tachycardia occurs in up to 14% of patients taking paliperidone [6] and can occur more than 24 hours after administration [7].

Here, we present the case of a patient with no known cardiovascular risk factors who developed tachycardia following initiation of risperidone, which improved with beta-blocker treatment, but subsequently worsened after paliperidone palmitate administration.

## 2. Patient Information

Mr. S was a 29-year-old male with major depressive disorder and generalized anxiety disorder brought to the emergency department due to depression and psychosis. He presented with low mood, anhedonia, paranoia, auditory and visual hallucinations, and insomnia. Per family, he had discontinued his home sertraline, mirtazapine, and hydroxyzine months earlier. Mr. S had no known past medical history, no known family history of psychiatric illness or cardiovascular illness, and no history of substance use.

Mr. S had a blood pressure of 135/68, heart rate (HR) of 69 beats per minute (bpm), temperature of 99.4°, and respiratory rate of 16 at presentation. Initial investigations, including complete blood count, complete metabolic panel, thyroid-stimulating hormone, urinalysis, urine drug screen, head CT, and lipid panel, were unremarkable except for LDL 152, total cholesterol 215, and white blood cell count 11.95. Electrocardiogram (EKG) demonstrated sinus rhythm with a ventricular rate of 77 BPM and QTc of 408 milliseconds.

## 3. Treatment/Management

Mr. S was admitted to the inpatient psychiatric unit with a diagnosis of major depressive disorder with psychotic features. His home sertraline 25 mg daily, mirtazapine 15 mg nightly, and atorvastatin 20 mg nightly were restarted. Trazodone 50 mg nightly as needed and hydroxyzine 50 mg every six hours as needed were prescribed for sleep and anxiety, respectively, while standing olanzapine was initiated for psychotic symptoms. However, the olanzapine was changed to risperidone starting at 2 mg daily on the morning of hospital day 4, with a goal of converting to paliperidone palmitate prior to discharge to improve postdischarge adherence. Paliperidone palmitate was initiated at 234 mg on hospital day 8, and a booster of 156 mg was administered on hospital day 16 in the late afternoon, followed by hospital discharge the following day. Though oral supplementation with risperidone or paliperidone is not required when initiating paliperidone palmitate using this approach [8], the treatment team titrated risperidone up to 3 mg twice daily on hospital day 8 and continued it at this dosage until the morning of hospital day 16, one day prior to hospital discharge. We are unaware of the team's rationale for this approach.

As Mr. S's psychiatric symptoms improved throughout his hospitalization, he unfortunately developed intermittent tachycardia. However, he did not report associated symptoms such as chest pain, palpitations, or lightheadedness. From presentation through hospital day 5, his HR was within normal limits, ranging from 69 to 99 bpm. However, it increased to 110 on hospital day 6, approximately 35 hours after receiving risperidone for the first time at 2 mg and 24 hours after a second dose of 1 mg (despite chart review, we were unable to determine why he received 1 mg instead of 2 mg at this time). After this HR reading, his risperidone dosage was increased to 2 mg twice daily and was then titrated up to 2 mg in the morning and 3 mg at night on hospital day 7, followed by further titration up to 3 mg twice daily on hospital day 8. During this risperidone titration period on hospital days 6-8, Mr. S's HR ranged from 84 to 121 bpm. Following stabilization of risperidone dosing at 3 mg twice daily on hospital day 8 and paliperidone palmitate 234 mg administration that evening, from hospital days 9 to 14, his HR steadily increased from 96 to 145 bpm. A notable change in HR occurred between the mornings of hospital days 9 and 10, as it rose from 96 bpm to 115 bpm (approximately 13 and 37 hours postpaliperidone palmitate administration, respectively). Given that median time to reach maximum plasma concentration (tmax) after a single intramuscular injection of paliperidone palmitate is 13 days and the fact that the patient's risperidone dosing was stable from hospital days 9 to 14, the gradual HR increase observed over that period suggests likely potentiation of tachycardia as paliperidone palmitate slowly dissolved and was hydrolyzed into paliperidone, which was then absorbed into the patient's systemic circulation [8].

**Figure 1:**
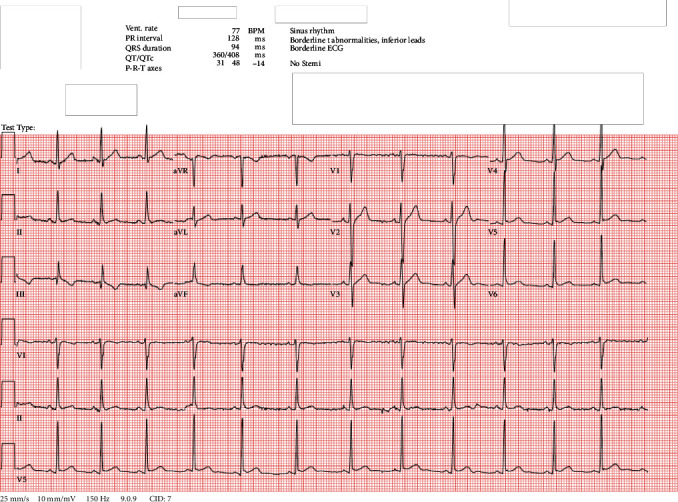
EKG from hospital day 1.

**Figure 2:**
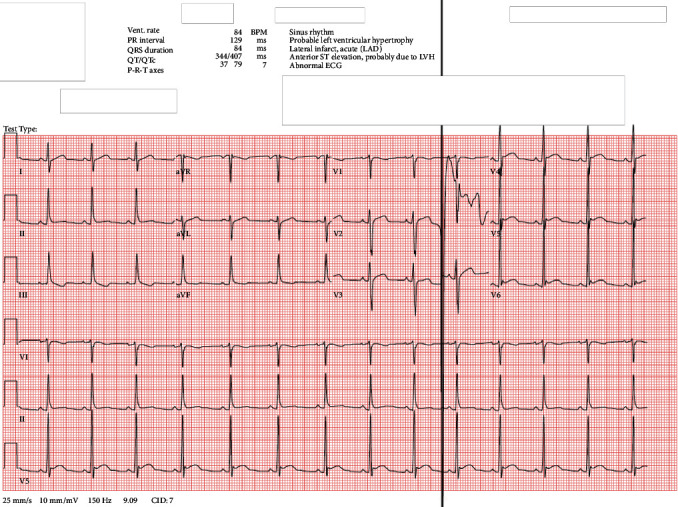
EKG from hospital day 15.

Propranolol 10 mg twice daily was initiated for his tachycardia following internal medicine consultation on the morning of hospital day 15, and that afternoon, EKG demonstrated sinus rhythm with HR of 84 bpm, down from 130 bpm that morning, which was measured prior to propranolol initiation (see Figure 1 for EKG obtained on hospital day 1 and Figure 2 for EKG obtained on hospital day 15). On hospital day 16, his HR ranged from 107 bpm in the morning (approximately 1 hour after morning propranolol and risperidone administration) to 99 bpm at night (5 minutes prior to nighttime propranolol administration). That same day, risperidone was discontinued following the morning dose and his second paliperidone palmitate injection was administered in the late afternoon. On the morning of hospital day 17, 14 hours after his second paliperidone palmitate injection and 23.5 hours after his last dose of oral risperidone, the patient's HR increased to 128 bpm. Later that day, with his psychiatric symptoms in good control, Mr. S was discharged home with outpatient psychiatric and medical follow-up. Unfortunately, we were not able to reach him to determine whether he continued on his discharge medication regimen or required medication changes to address ongoing tachycardia. There were also no records of follow-up in our hospital system's electronic medical record.

**Figure 3:**
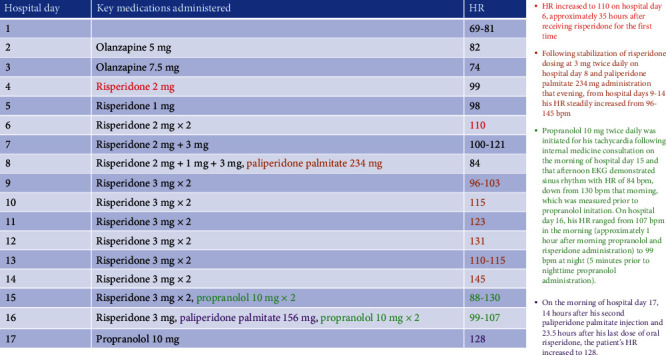
Hospital course graphic.

More information about the treatment course can be visualized in Figure 3. Of note, the patient's BP was consistently measured less than or equal to 140/90, except for 3 aberrant readings that occurred both in the presence and absence of tachycardia.

## 4. Discussion

Prompt identification and management of tachycardia are essential in patients with serious mental illness being treated with antipsychotics, since many already have risk factors for cardiovascular disease. Left untreated, prolonged tachycardia can contribute to cardiomyopathy and heart failure [9]. This case contributes to the limited previous literature indicating an association, in some patients, between risperidone [3–5, 7, 10–12] and tachycardia, which can be further exacerbated by paliperidone palmitate [3, 4, 6, 7, 10–12]. The following temporal relationships between medication administrations and Mr. S's tachycardia lend support to this conclusion for his case. His HR increased substantially approximately 35 hours following risperidone initiation, as well as approximately 37 hours after the first paliperidone palmitate injection. Another large increase occurred 14 hours after the second paliperidone palmitate administration, which was also nearly 24 hours since his final dose of risperidone. These time frames are consistent with past literature indicating tachycardia can occur many hours after risperidone/paliperidone initiation [4, 5], dosage increase [12], or overdose [7].

We also considered whether other administered medications capable of inducing tachycardia were the source of Mr. S's tachycardia. Sertraline is associated with tachycardia in <2% of patients [13]. However, it was discontinued on hospital day 9, and Mr. S had been taking this for the first five days of his hospitalization without experiencing tachycardia. Olanzapine is associated with tachycardia in 3% of patients [14] but was discontinued on hospital day 3. Trazodone is associated with tachycardia in <2% of patients [15] and was administered only on hospital days 5 and 6.

Potential interactions between some of Mr. S's other medications may lead to tachycardia, but none were particularly likely. Interactions between the serotonergic medications he was taking (sertraline and trazodone) can cause serotonin syndrome, but Mr. S lacked signs of serotonin syndrome. These medications, as well hydroxyzine, may contribute to prolongation of the QTc interval, which can cause tachycardia, but the patient's QTc was normal at 408 ms on hospital day 15. Dopamine-blocking medications such as olanzapine, paliperidone, and risperidone may cause neuroleptic malignant syndrome (NMS), but Mr. S lacked signs of NMS. No other potential interactions between medications Mr. S was taking were identified.

When applying the Naranjo Adverse Drug Reaction (ADR) Probability Scale [16], an instrument designed to assess causality for adverse drug reactions, we found an ADR score of 7 for both risperidone and paliperidone palmitate, indicating a probable link with this patient's tachycardia. Unfortunately, we are not able to comment on the type of tachycardia Mr. S experienced, since he did not undergo continuous cardiac monitoring. Though he did undergo two EKGs during his admission, both of which demonstrated sinus rhythm with normal HR, the first occurred at the start of his admission and the second occurred after propranolol was initiated. The fact that another EKG was not obtained in between these two highlights the importance of promptly obtaining an EKG to help guide treatment should a patient develop persistent tachycardia.

One future consideration would be to use Carbamazepine to more rapidly decrease drug levels in patients with unwanted side effects associated with long-acting injections. In particular, Carbamazepine can be used to expedite metabolism of risperidone through induction of CYP3A4 [17].

This case provides further evidence of an association between risperidone/paliperidone palmitate and tachycardia occurring several hours, or even more than 24 hours, postadministration. With this in mind, it is imperative that psychiatrists regularly monitor vital signs in patients recently initiated on these medications to identify and appropriately manage this potentially harmful side effect.

## Data Availability

The deidentified patient data used in this case report are available from the corresponding author upon request.
